# Resveratrol Inhibits the Epithelial Sodium Channel via Phopshoinositides and AMP-Activated Protein Kinase in Kidney Collecting Duct Cells

**DOI:** 10.1371/journal.pone.0078019

**Published:** 2013-10-24

**Authors:** Kelly M. Weixel, Allison Marciszyn, Rodrigo Alzamora, Hui Li, Oliver Fischer, Robert S. Edinger, Kenneth R. Hallows, John P. Johnson

**Affiliations:** 1 Renal-Electrolyte Division, Department of Medicine, University of Pittsburgh, Pittsburgh, Pennsylvania, United States of America; 2 Department of Cell Biology, University of Pittsburgh, Pittsburgh, Pennsylvania, United States of America; 3 Biology Department, Washington and Jefferson University, Washington, Pennsylvania, United States of America; University of Houston, United States of America

## Abstract

Resveratrol, a naturally occurring phytoalexin, has reported cardioprotective, anti-inflammatory, chemopreventative and antidiabetic properties. Several studies indicate the multiple effects of resveratrol on cellular function are due to its inhibition of class 1A phosphoinositide 3-kinase (PI3K) mediated signaling pathways, but it also activates AMP-activated protein kinase (AMPK). As sodium transport in the kidney via the Epithelial Sodium Channel (ENaC) is highly sensitive to changes in phosphoinositide signaling in the membrane and AMPK, we employed resveratrol to probe the relative effects of phosphatidylinositol species in the plasma membrane and AMPK activity and their impact on ENaC activity in mouse cortical collecting duct (mpkCCD_c14_) cells. Here we demonstrate that resveratrol acutely reduces amiloride-sensitive current in mpkCCD_c14_ cells. The time course and dose dependency of this inhibition paralleled depletion of the PI(3,4,5)P_3_ reporter (AKT-PH) in live-cell microscopy, indicating the early inhibition is likely mediated by resveratrol's known effects on PI3K activity. Additionally, resveratrol induces a late inhibitory effect (4–24 hours) that appears to be mediated via AMPK activation. Resveratrol treatment induces significant AMPK activation compared with vehicle controls after 4 h, which persists through 16 h. Knockdown of AMPK or treatment with the AMPK inhibitor Compound C reduced the late phase of current reduction but had no effect on the early inhibitory activity of resveratrol. Collectively, these data demonstrate that resveratrol inhibits ENaC activity by a dual effect: an early reduction in activity seen within 5 minutes related to depletion of membrane PIP_3_, and a sustained late (4–24 h) effect secondary to activation of AMPK.

## Introduction

The epithelial Na^+^ channel (ENaC) plays a key role in the regulation of Na^+^ absorption in the distal nephron (reviewed in [1], [2]). While the majority of filtered Na^+^ is reabsorbed along the length of the nephron, the fine-tuning of Na^+^ absorption in the distal segment relies largely on the number of active ENaC channels in the apical membrane of the principal cells. Abnormalities of ENaC function have been linked to disease states including hypertension and pseudohypoaldosteronism (PHA) [3]–[5]. The channel is important in physiologic regulation in kidney, lung and colon. Therefore, it’s activity is subject to a number of regulatory controls. Increasing the surface density of ENaC, and therefore Na^+^ absorption, is mediated by a variety of hormonal factors such as mineralocorticoids, vasopressin and insulin. Several mechanisms of hormone-induced sodium transport involve phosphoinositide (PI) synthesis and metabolism and thus intersect with the trafficking pathways that alter surface density of ENaC. Indeed, studies from our laboratory and others support the hypothesis that modulation of the kinases that alter the compartmentalization of PIs may stimulate channel insertion and regulate channel endocytosis.

We have demonstrated that ENaC is retrieved into the cell as a ubiquitinated protein via clathrin-mediated endocytosis with epsin as the adaptor protein [6]. This process is in part regulated by phosphatidylinositol 4,5-bisphosphate, PIP_2_ [7]. Following retrieval, a substantial number of the channels are deubiquitinated and returned to the apical membrane by the recycling pathway, a process also influenced by enzymes that synthesize and metabolize PIs, including both PIP_2_ and PI(3,4,5)P_3_. Phosphatidylinositide 3-OH kinase (PI3-K) is a critical component of many signaling pathways regulating ENaC activity. Aldosterone and insulin have both been shown to increase the activity of PI3-K, resulting in activation of signaling pathways that lead to an increase in ENaC expression at the apical membrane [8]–[10]. In addition, there is strong evidence that both PIP_2_ and PI(3,4,5)P_3_ can directly bind to ENaC and influence channel open probability, P_o_, another mechanism of altering overall ENaC function [11]–[14]. PI3-K has also been identified as one of the central pathways affected by resveratrol, a naturally occurring polyphenolic compound that has a wide range of anti-inflammatory, antioxidant and cytoprotective effects (reviewed in [15]–[18]). Resveratrol is also known to prevent cardiac hypertrophy and attenuate hypertension in hypertensive rats or mice [19]–[21]. We therefore proposed that resveratrol might have significant effects on ENaC activity mediated through the PI3-K pathway and would be a useful agent for probing the interactions between PIs and ENaC activity.

Interestingly, resveratrol is also thought to have cytoprotective and anti-aging effects that are mediated via activation of the metabolic sensor, AMP-activated protein kinase (AMPK) [22]–[24]. This kinase has also been identified as an inhibitor of ENaC, decreasing channel number through regulation of ubiquitination [25]. Resveratrol, therefore, lies squarely across two significant pathways of ENaC regulation, PI3-K and AMPK, and should have significant effects on ENaC activity potentially through effects on PIP_2_, PI(3,4,5)P_3_ and/or AMPK levels. We have used the agent to probe the intersection of these pathways in mpkCCD_c14_ cells, a native ENaC culture model. We describe a complex but profound response, mediated initially through effects on PI3-K and a sustained later effect mediated in part by activation of AMPK. These effects may contribute to the antihypertensive effects of resveratrol recently described in animal models [19].

## Materials and Methods

### Reagents

All reagents and chemicals used were purchased from Sigma unless otherwise noted. Phospho-Thr172-AMPKα antibody was purchased from Cell Signaling Technology (Danvers, MA), AMPKα1 antibody was obtained from Upstate Millipore. Compound C (6-[4-[2-(1-Piperidinyl)ethoxy]phenyl]-3-(4-pyridinyl)-pyrazolo[1,5-a]pyrimidine) was obtained from Enzo Life Sciences (Plymouth Meeting, PA). HRP-conjugated anti-mouse and anti-rabbit antibodies were purchased from GE Healthcare (Piscataway, NJ). The PIP_2_ reporter, the PH domain from PLCδ1 cloned into pEGFP-N1, was the generous gift of Dr. T Balla (National Institutes of Health). The reporter Akt-PH-11 was kindly provided by Dr. J. Stockand (University of Texas Health Sciences Center).

### Cell culture

Mouse cortical collecting duct (mpkCCD_c14_) cells obtained from Alain Vandewalle (INSERM)[26] were cultured in defined media containing FBS, antibiotics, and other hormones and nutrients as previously described [26]. The cells were seeded at confluent density onto 6.5-mm or 12-mm diameter Transwells (Costar, Corning, NY) and cultured for at least 5 days prior to experimentation to allow for polarization and transepithelial resistance development.

### Short-circuit current measurements

mpkCCD_c14_ cells cultured on filter supports were mounted in modified Ussing chambers, and short circuit currents (*I*_sc_) were measured with an automatic voltage clamp. Cells were recorded approximately 30 min prior to addition of resveratrol or vehicle control to apical and basolateral cell surfaces. The *I_sc_* tracing was recorded for an additional 45 minutes. Amiloride (10 µM) was added at the end of each experiment to derive the amiloride-sensitive component of the short circuit current. Typically, ≥90% of the total *I*_sc_ was inhibited after amiloride addition. The bathing Ringer's solution was composed of 120 mM NaCl, 25 mM NaHCO_3_, 3.3 mM KH_2_PO_4_, 0.8 mM K_2_HPO_4_, 1.2 mM MgCl_2_, 1.2 mM CaCl_2_, and 10 mM glucose. The chambers were constantly gassed with a mixture of 95% O_2_, 5% CO_2_ at 37°C, which maintained the pH at 7.4. To determine the effect of resveratrol treatment for up to 24 hours, transepithelial Na^+^ current across mpkCCD_c14_ cell monolayers cultured on 6.5-mm permeable supports (Corning; Lowell, MA, USA) and kept in culture media was calculated using Ohm's law as the quotient of transepithelial potential difference to transepithelial resistance under open circuit conditions using an EVOM volt-ohm meter with dual Ag/AgCl pellet electrodes (World Precision Instruments; Sarasota, FL, USA) to measure voltage and resistance.

### Generation of stable AMPK-α1 shRNA knockdown and nontargeting control mpkCCD_c14_ cells

Two complementary primers were used to generate a hairpin targeting mouse AMPK-α1 (GATCCCC**GCTGTGGCTCACCCAATTA**TTCAAGAGATAATTGGGTGAGCCACAGCTTTTTC) at nucleotide 517 (517; relevant hairpin sequence in **bold**), along with a control hairpin that does not target any known mammalian gene (GATCCCC**GCGCGCTTTGTAGGATTCG**TTCAAGAGACGAATCCTACAAAGCGCGCTTTTTC) (MamX). The oligonucleotides were annealed and first ligated into the pEN vector (shuttle or ENtry vector; obtained from ATCC) and then transferred to the shRNA-expressing lentivirus vector pDSL_hpUGIP (ATCC) by LR-clonase (Invitrogen). Recombinant lentiviruses were generated by co-transfection of these individual plasmids with a mixture of packaging vectors (pCMV-ΔR8.2 and pCMV-VSV-G; Addgene) into HEK 293T packaging cells using Lipofectamine 2000. Viral supernatants were harvested 72 h after transfection. Viral particles (∼1×10^7^/prep were concentrated using an Amicon Ultra 50 filter unit (Millipore #UFC905024). mpkCCD_c14_ cells grown in a 6-well plate were transduced with lentiviral particles at a multiplicity of infection of ∼1. After 2 weeks of selection in puromycin (2 µg/ml), cells were expanded and plated on 0.33-cm^2^ Costar Transwell filters (#3470) and allowed to grow to high resistance before use in experiments 5–9 d later.

### Immunoblotting and quantitation of AMPK activation

mpkCCD_c14_ cells grown on 12-mm Transwell filters were treated with resveratrol for the indicated time intervals, washed twice in ice-cold PBS and then lysed as previously described [27]. After pelleting the lysates and measuring protein concentrations of the supernatants, equal amounts of cellular protein per lane were loaded on a 4–12% gradient gel (NuPage, Invitrogen, Carlsbad, CA) and subjected to SDS-PAGE followed by transfer to a nitrocellulose membrane. Membranes were blocked in 5% bovine serum albumin, and immunoblotting was performed using AMPK-α1 (1∶1000), pThr^172^-AMPKα (1∶1000), or β-actin (1∶5000) primary antibodies. Secondary donkey anti-rabbit or sheep anti-mouse antibodies were used at 1∶5000. Intensities of all relevant bands corrected for local background intensity on the blot were quantitated using a VersaDoc Imager with Quantity One software (Bio-Rad). To measure AMPK activation in the cell lysates, the pThr^172^-AMPKα band signal in each lane was normalized to the intensity of β-actin as a loading control.

### Transfections and live-cell imaging

For transfection, mpkCCD_c14_ cells were seeded at 30% confluence onto 0.17-mm Delta T glass-bottomed dishes (Bioptechs, Inc) and cultured for 16–24 h to allow cells to adhere. The cells were then transferred to unsupplemented Opti-MEM media (Invitrogen) and immediately transfected with 1.5 ug DNA and Lipofectamine 2000™ reagent (Invitrogen) according to the manufacturer's instructions. After 5-h incubation at 37°C, the transfection solution was replaced with DMEM/F12 medium and cells were returned to incubation. Cells were imaged 24–26 h post-transfection. Transfected cells were imaged in Opti-MEM media supplemented with 5% FBS (Atlanta Biologicals) and 40 mM HEPES and maintained at 35°C during imaging. Epiflourescent time-lapse images were taken on an Olympus IX81 microscope (Melville, NY) equipped with a RETIGA EXI (QImaging) and XENON lamp (Olympus) with appropriate filter combinations for GFP. Time-lapse images were taken with a PlanApo 60× oil immersion objective (1.4 NA) and Metamorph imaging software. Images were taken at 5-min intervals for 30–90 min. Resveratrol was added directly to the imaging media to a final concentration of 100–200 µM, with maximal effect at 200 µM. Images were exported in a tag-information-file format (TIFF), and contrast was adjusted in Adobe® Photoshop® (Adobe, Mountain View, CA). Image analysis was performed using ImageJ as described [28].

### Statistics

Statistical analyses were performed using StatView (SAS Institute Inc, Cary, NC). Analysis of variance was used to compare data obtained from different batches of cell monolayers for short circuit current experiments. For other biochemical experiments, statistics were performed using Student's *t* tests, as described in each figure legend. In all cases, *p* values <0.05 were considered significant.

## Results

### Resveratrol acutely inhibits amiloride-sensitive currents in polarized mpkCCD_c14_ cells in a PI3K mediated-manner

**Figure 1:**
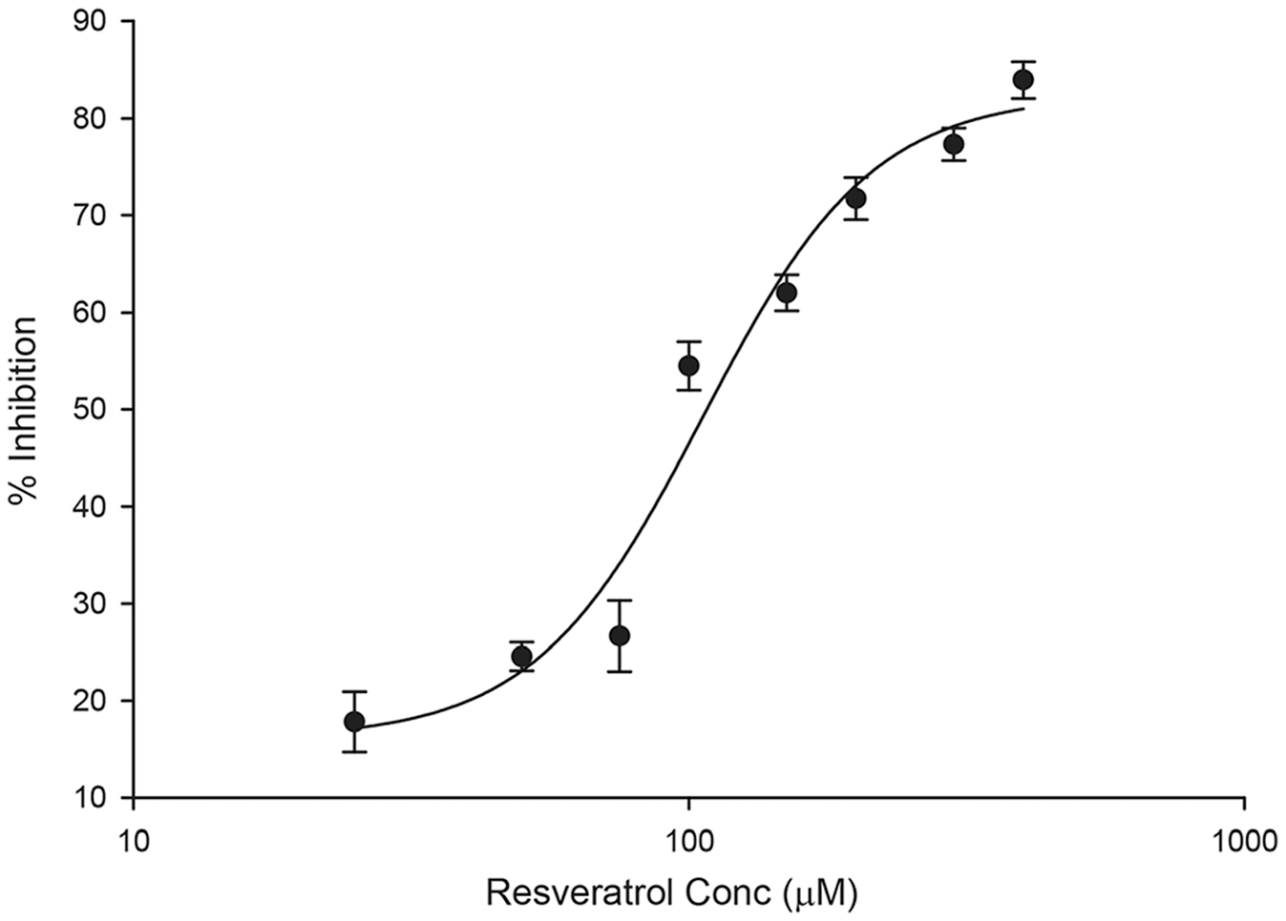
Resveratrol inhibits amiloride-sensitive currents in polarized mpkCCD_c14_ cells in a dose-dependent. Polarized mpkCCD_c14_ were mounted in modified Ussing chambers and treated with increasing concentrations (25, 50, 75, 100, 150, 200, 300, 400 µM) resveratrol to both apical and basal compartments. Currents were monitored for 30 minutes. 10 µM amiloride was added to the apical compartment at the end of the experiment to determine net Na^+^ transport through ENaC. The amiloride-sensitive % current inhibition was plotted versus concentration (mean ± SEM) and fitted using a sigmoid, 4 parameter curve. The effective IC_50_ dose is 107 µM. N = 4 for each concentration. Amiloride abolished greater than 95% of the starting current in all conditions tested.

To examine the effects of resveratrol on sodium transport, short-circuit currents were measured in mpkCCD_c14_ cells mounted in a modified Ussing chambers following treatment with increasing concentrations of resveratrol or vehicle in **Figure 1**). Current and resistance was monitored for 30 minutes, after which 10 µM amiloride was added to the apical compartment to confirm the net Na^+^ transport via ENaC. Resveratrol inhibited Na^+^ current via ENaC in a dose-dependent manner, with an IC_50_ does of 107 µM, (**Figure 1**). The inhibitory effect begins to level off at 200 µM and this concentration was used for subsequent experimental analysis (**Figure 1**). The transepithelial resistance measurements increased over the 30 minute time course at all doses (data not shown) consistent with decreasing ENaC activity.

**Figure 2:**
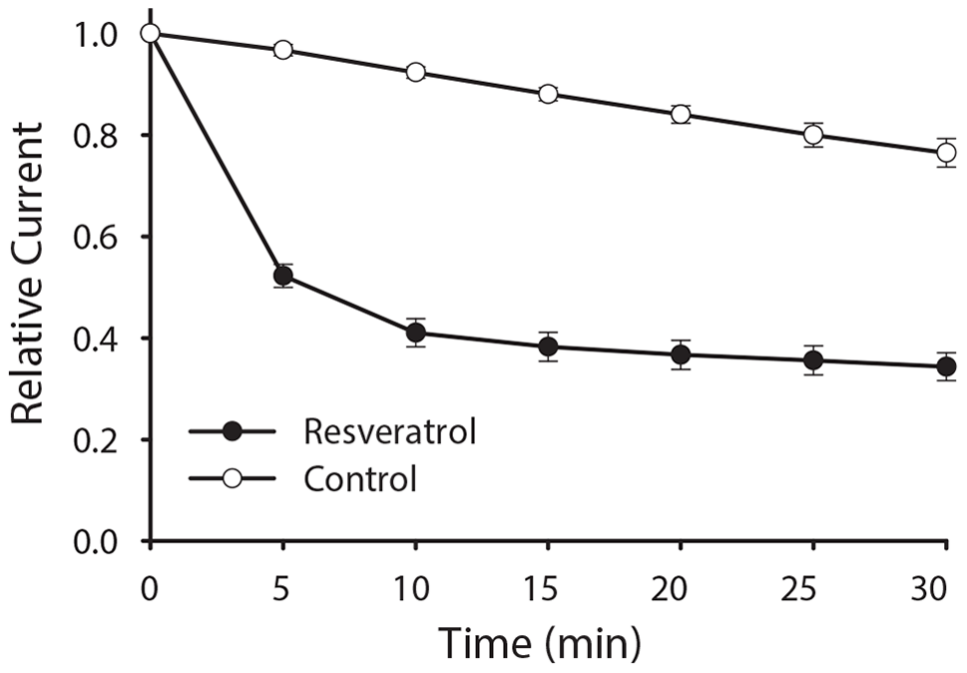
Resveratrol acutely inhibits ENaC currents in polarized mpkCCD_c14_ cells. Polarized mpkCCD_c14_ were mounted in modified Ussing chambers and treated with 200 µM resveratrol (closed circles) or vehicle control (open circles) to both apical and basal compartments. Currents were monitored over the next 30 minutes. Amiloride was added to the apical cell surface at the end of the experiment to determine net Na^+^ transport through ENaC. The relative amiloride-sensitive current (mean ± SEM) is graphically shown over five minute intervals. N = 7 for control and 13 for resveratrol, *p*<0.001 at each time point.

Similar experiments were performed to examine the time course of resveratrol induced inhibition of sodium transport in mpkCCD_c14_ cells. Resveratrol reduced Na^+^ current by 50% within 5 minutes, an effect that persisted for the entire 30 minute recording (**Figure 2**).

**Figure 3:**
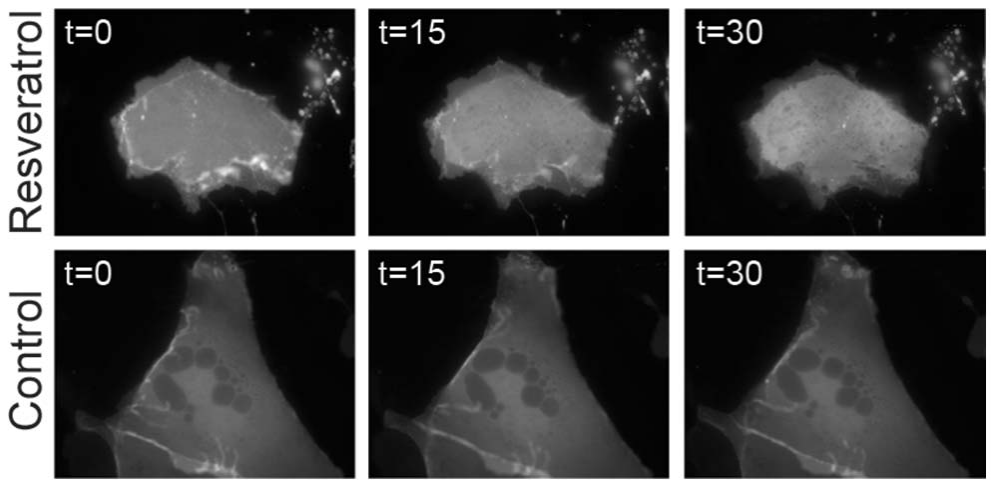
Resveratrol induces redistribution of PI [3], [4], [5]P_3_ reporter GFP-AKT-PH in mpkCCD_c14_ cells. Cultured mpkCCD_c14_ cells were transfected with GFP-AKT-PH and examined by live-cell epifluorescence microscopy. Addition of resveratrol (*upper panels*) caused a rapid redistribution of PI [3], [4], [5]P_3_ from the plasma membrane whereas control-treated cells showed little change (*lower panels*). Representative data are shown from four independent trials and representative of identical observations.

**Figure 4:**
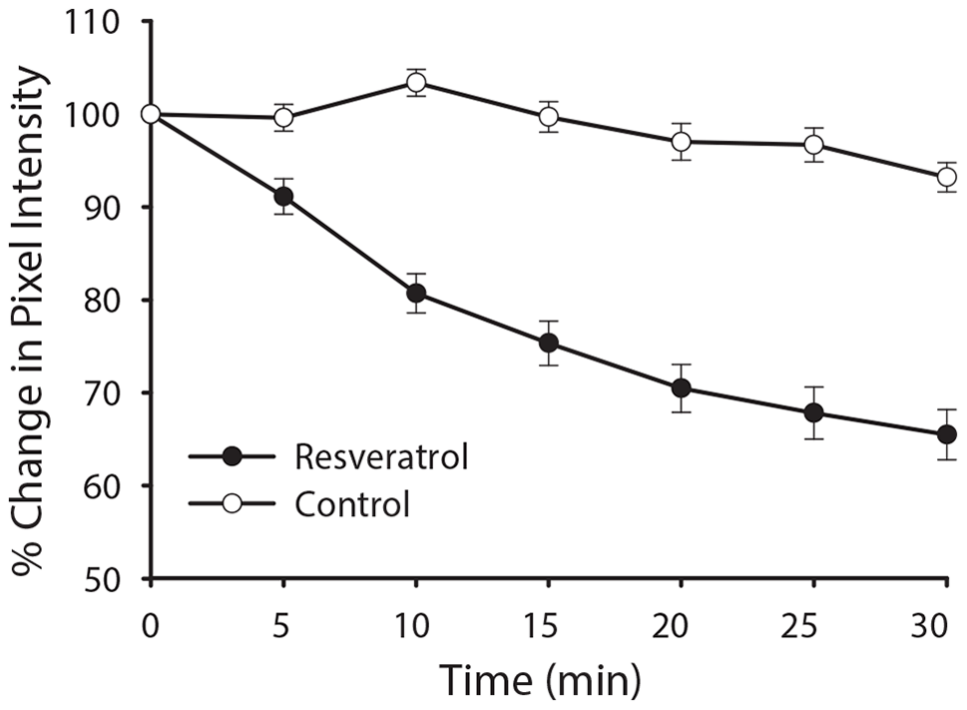
Time course of PI [3], [4], [5]P depletion from plasma membrane of mpkCCD_c14_ cells parallels change in ENaC current. The percent change of average pixel intensity of GFP-AKT-PH in cells treated with resveratrol (closed circles) versus vehicle treatment (open circles) is plotted against time. Decay of plasma membrane PI [3], [4], [5]P levels within 10 min of application. Graph shows mean ± SEM; N = 10; *p<0.05).

Resveratrol has been demonstrated to inhibit PI3-K by competing with ATP for the catalytic site [29]. Previous studies examining the role of PI3-K signaling show that inhibiting PI3-K signaling pathways rapidly decreases ENaC activity [12], [30]. Therefore, we reasoned that the immediate effect may be mediated by resveratrol's known inhibition of PI3-K. To test this hypothesis, mpkCCD_c14_ cells were transfected with the PI[3], [4], [5]P_3_ reporter, GFP-AKT-PH and examined by live-cell epifluorescence microscopy. Addition of resveratrol (**Figure 3**, *upper panels*) causes a rapid redistribution of PI[3], [4], [5]P_3_ away from the plasma membrane, as compared with control vehicle-treated cells (**Figure 3**, *lower panels*). Vehicle treatment was used to evaluate decreases in fluorescence emissions over time due to photobleaching. Furthermore, the observed change in mean pixel intensity of GFP-AKT-PH at the plasma membrane parallels the acute change in short circuit current (**Figure 4** compared to **Figure 2**), with a decay of plasma membrane PI[3], [4], [5]P_3_ levels that is maximal within 10 min of application. These observations support the hypothesis that the early effects of resveratrol on Na^+^ transport are mediated by modulation of the PI3K signaling pathway. Resveratrol had no effect on PIP_2_ distribution at the plasma membrane as assessed by similar studies using a PIP_2_ marker, GFP-PLCδ-PH domain (data not shown).

### AMPK mediates the late inhibitory effect of resveratrol on amiloride-sensitive currents in polarized mpkCCD_c14_ cells

**Figure 5:**
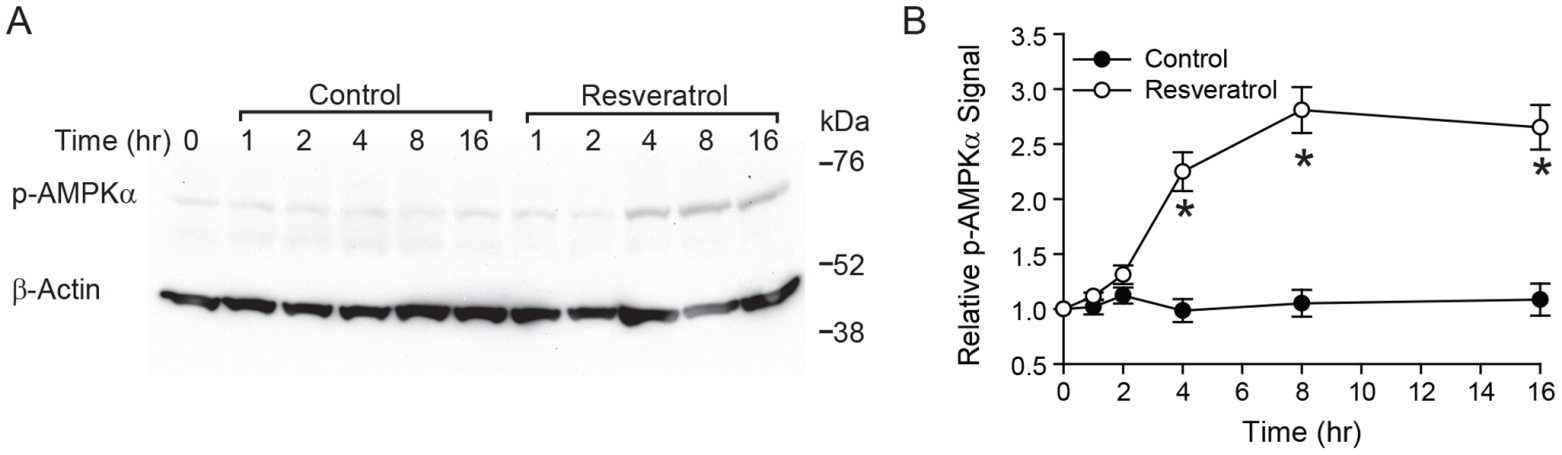
Resveratrol treatment activates AMP-activated protein kinase (AMPK) in mouse cortical collecting duct mpkCCD_c14_ cells. Polarized mpkCCD_c14_ cells were fed with media containing resveratrol or vehicle control for the times indicated. Cell extracts (50 µg protein) were separated on a 4–12% SDS gel. (*A*). Representative immunoblot analysis of the time-dependence of resveratrol-mediated AMPK activation, as measured by immunoblotting of phosphorylated AMPKα (p-AMPKα) in polarized mpkCCD_c14_ cells. (*B*). Density of p-AMPKα bands were quantified and normalized to protein loading using β-actin as control, and means ± SEM were plotted (*n* = 3), *p<0.01 relative to vehicle control at same time point.

Previous studies have indicated that AMPK inhibits ENaC in both oocytes and mouse polarized kidney epithelial cells [25], [31]. As AMPK is a target of resveratrol in several model systems, we investigated whether resveratrol could modulate the AMPK-mediated pathways that inhibit ENaC. Polarized mpkCCD_c14_ cells were fed with media containing resveratrol or vehicle control (ethanol) for 16 h. At various time intervals cell extracts were examined by immunoblotting for phosphorylated AMPKα (p-AMPKα) to measure activation of AMPK in the presence or absence of resveratrol. Resveratrol treatment induced a significant activation of AMPK compared with vehicle controls beginning at 4 h post-treatment and continued for at least 16 h. (**Figure 5A**) Densitometric analysis demonstrates a ∼2.5-fold increase in AMPK activation in resveratrol-treated cells at times ≥4 h (**Figure 5B**). These results demonstrate time dependence in resveratrol-mediated AMPK activation.

**Figure 6:**
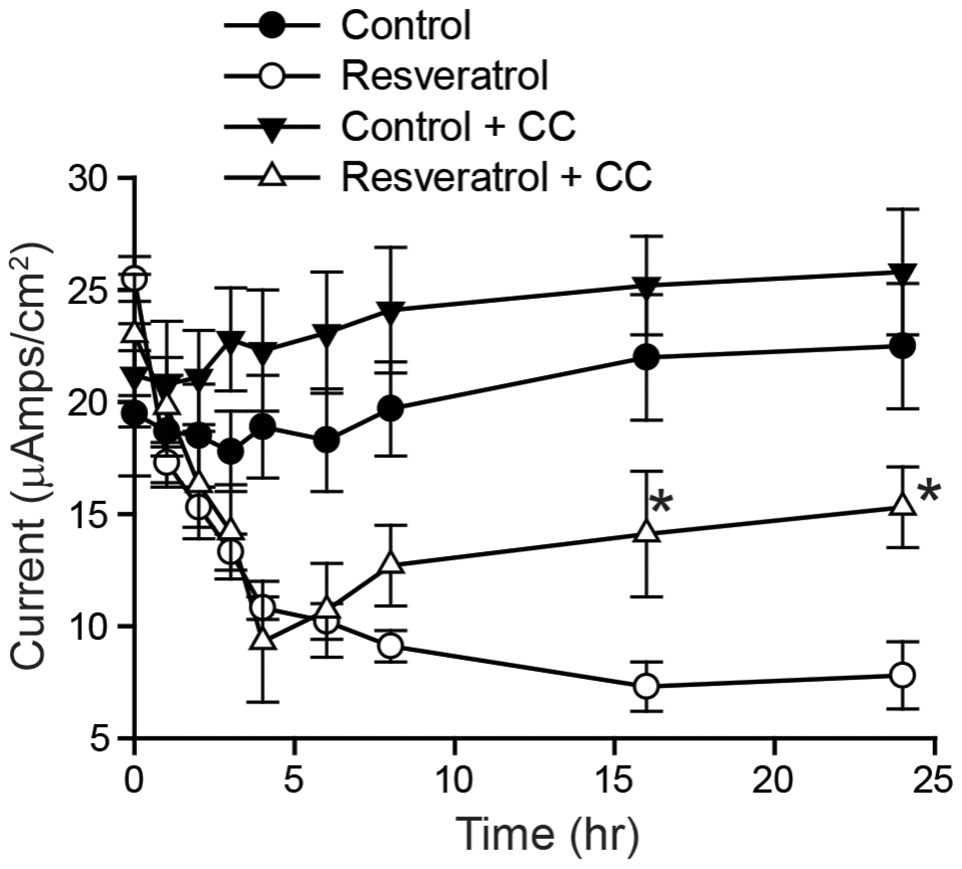
Acute inhibition of AMPK with Compound C prevents the late inhibitory effect of resveratrol on amiloride-sensitive currents in polarized mpkCCD_c14_ cells. Polarized mpkCCD_c14_ cells were pretreated with media containing 50 µM Compound C or vehicle (DMSO) and incubated for 30 min to allow for AMPK inhibition. In groups pretreated with Compound C, this drug was present throughout the resveratrol treatment. Equivalent short circuit currents (*I_sc_*, µA/cm^2^) were measured at the times indicated. Values are mean ± SEM of 3 independent experiments with 4 samples per group. *P<0.01 relative to cells treated with resveratrol alone at same time point.

To investigate whether the late inhibitory effect of resveratrol is due to AMPK activity, mpkCCD_c14_ cells were pretreated with the AMPK inhibitor Compound C or vehicle (DMSO) for 30 min to allow for AMPK inhibition before resveratrol treatment (**Figure 6**). Pretreatment with Compound C significantly reduced the late (8–24 h) inhibitory effect of resveratrol on amiloride-sensitive currents in mpkCCD_c14_ cells but did not affect the early (0–6 h) inhibitory effect of resveratrol. Current values for control cells (no resveratrol) with or without Compound C were not statistically different. These results demonstrate that acute inhibition of AMPK abrogates the late inhibitory effect of resveratrol on ENaC current.

**Figure 7:**
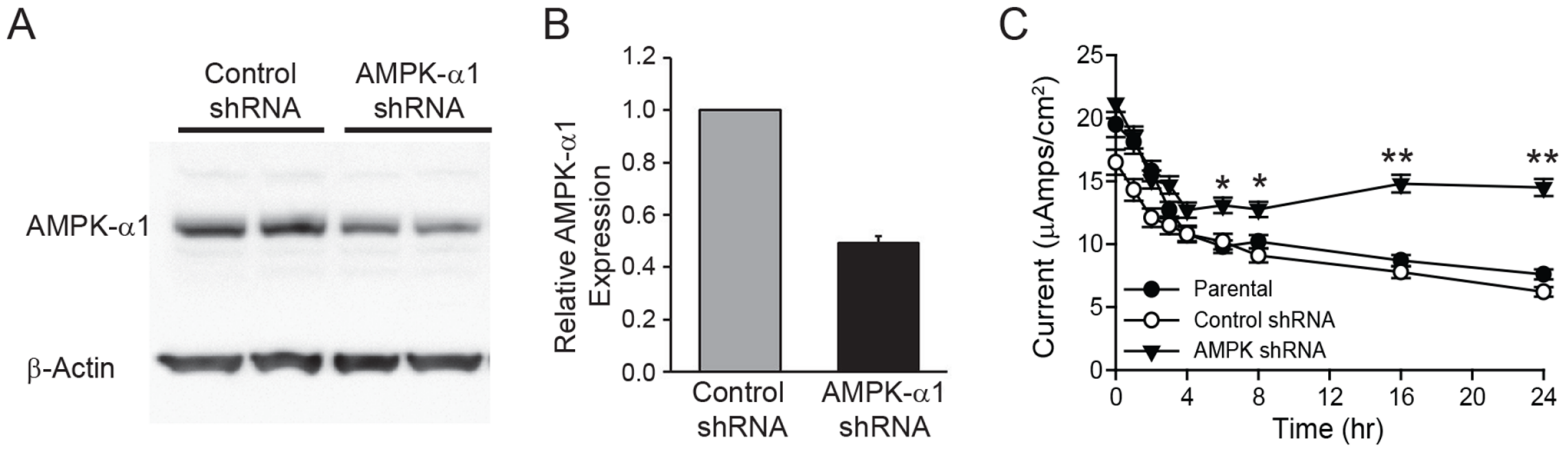
Knockdown of AMPK reduces the late inhibitory effect of resveratrol on amiloride-sensitive currents in mpkCCD_c14_ cells. mpkCCD_c14_ cells were lentivirally transduced to express shRNAs directed against AMPKα1 or a control shRNA that does not target any known mammalian gene. (A) Cell extracts (50 µg protein) were separated on a 4-12% SDS gel. Representative immunoblot analysis showing the extent of AMPK knockdown. (*B*) Densities of AMPK bands were quantified and normalized to protein loading using β-actin as control, and means ± SEM were plotted relative control shRNA. (C) Untransduced (parental), AMPKα1 shRNA or control shRNA transduced mpkCCD_c14_ cells were treated with resveratrol starting at time 0. Equivalent short circuit currents (*I_sc_*, µA/cm^2^) were measured for the times indicated. No significant difference was observed in current values between untransduced cells (no shRNA) and cells transduced to express control shRNA. Values are mean ± SEM of 3 independent experiments with 4 samples per group. *p<0.05, **p<0.01 relative to control shRNA at same time point.

To test more directly the role of AMPK in mediating the effects of resveratrol observed between 4–24 h, we downregulated AMPKα1 protein expression in mpkCCD_c14_ cells by RNA interference and examined the effects of resveratrol. Knockdown of AMPKα1 was confirmed by immunoblotting for AMPKα1 expression (**Figure 7A**) in mpkCCD_c14_ cells that had been stably transfected with either control or AMPKα1 shRNA. Densitometric analysis (**Figure 7B**) demonstrated that AMPKα1 shRNA-mediated knockdown reduced protein expression by ∼50% relative to control shRNA. AMPKα1 knockdown significantly reduced the late (4–24 h) inhibitory effect of resveratrol on amiloride-sensitive currents in mpkCCD_c14_ cells (*p*<0.01 relative to control shRNA at same time point) but did not affect the early (0–4 h) inhibitory effect of resveratrol, (**Figure 7C**). No significant difference was observed in current values between parental cells (no shRNA) and cells transfected with control shRNA. Interestingly, the inhibition of Na^+^ current that is observed in the first 30 min post-resveratrol treatment is preserved in the knockdown, indicating that this effect is independent of AMPK.

## Discussion

Resveratrol is a naturally occurring polyphenolic compound, a phytoalexin found in various vegetal dietary sources, which has been described to have a wide variety of cytoprotective and chemoprotective effects [24], [32]. Resveratrol has been described as mimicking the biologic effects of caloric restriction and having antidiabetic as well as antioxidative and anti-inflammatory effects at the cellular level [22]–[24], [33]. Furthermore, it has been shown to have protective effects in cardiovascular and renal diseases [19]–[21], [33], [34] and to extend lifespan in lower eukaryotes [18]. These diverse effects have been linked to actions on a number of cellular pathways. For example, its antidiabetic effects have been linked to its action as a class 1A PI3-K inhibitor and downstream effects on PKB/Akt [29], [35], [36]. Anti-aging and caloric restriction actions have been linked to activation of Sirtuin-family proteins, and anti-inflammatory actions have been linked to inhibition of cyclo-oxygenase-1 [33], [35]-[37]. Effects on age-related phenotypes and on cardiovascular and renal diseases have been linked to activation of the calcium/calmodulin-dependent kinase kinase β (CamKKβ)-AMPK pathway as well as enhancement of AMPK activity through the LKB1/AMPK pathway [24]. Clearly, the beneficial effects of resveratrol result from its ability to influence distinct signaling pathways separate from its anti-oxidative properties. The cardioprotective effects of resveratrol have been attributed to its influence on endothelium-dependent smooth muscle relaxation as well as cardiac contractile and mitochondrial function [20]. Resveratrol improves survival, hemodynamics and energetics in a rat model of hypertension leading to heart failure. However, the cellular targets of resveratrol also include pathways that influence sodium and water transport in the distal kidney, physiological partners in regulating volume and blood pressure.

The epithelial sodium channel, ENaC, is tightly regulated in both number (*N*) at the apical membrane, and open probability (*P_o_*) by a variety of hormonal and cellular regulatory pathways (reviewed in [1], [2]). Among those, are several of the pathways impacted by resveratrol, particularly the PI3-K pathway and the energy sensor AMPK [12], [25], [38], [39]. PI3-K regulates the channel by controlling the levels of ENaC at the apical membrane and modulating channel *P_o_* via a direct interaction with the channel. Inhibition of PI3-K activity in ENaC-expressing cells has been shown to decrease channel activity in parallel with decreases in ENaC expression at the plasma membrane [12]. AMPK has been shown to participate in regulation of *N* at the apical membrane by regulation of the ubiquitin ligase Nedd4-2 and stimulating retrieval of the channel from the membrane [25]. We speculated that the cardioprotective and renal protective effects ascribed to resveratrol could, in part, be mediated through an action on ENaC. Accordingly, we examined the effects of resveratrol on ENaC activity in natively expressing cortical collecting duct cells in culture, with particular attention to the effects on PI3-K pathway and AMPK activation. We found that resveratrol profoundly inhibits ENaC activity in mpkCCD_c14_ cells in a rapid and sustained manner. The early effects appear to be mediated by PI3-K inhibition while the sustained effects require the activation of AMPK.

Addition of resveratrol to mpkCCD_c14_ cells resulted in a rapid and dose-dependent decline in Na^+^ current over a period of minutes (**Figure 1** **and** **2**). Na^+^ current was reduced by greater than 50% within 5-10 minutes of resveratrol addition and remained suppressed for a period of hours as long as resveratrol remained present (**Figure 2** **and** **5**). The acute decline in current over a period of minutes paralleled the decline in PI [3], [4], [5]P_3_ levels observed via live-cell microscopy (**Figure 4**). This is precisely the same pattern both in time course and relative current inhibition described by Staruschenko and colleagues [12], [40] with the use of the PI3-K inhibitor LY294002. The rapid decline in PI [3], [4], [5]P_3_ levels and Na^+^ current following addition of resveratrol is consistent with the inhibition of PI3-K activity described in other tissues [21], [29] and also suggests a significant effect of PI [3], [4], [5]P_3_ on maintaining ENaC activity under basal culture conditions in CCD cells. The studies documented here are consistent with this being a plasma membrane pool of PI [3], [4], [5]P_3_, but cannot rule out the potential for changes in sub-membrane pools of PI [3], [4], [5]P_3_ participating in this phenomenon. The rapidity of the decline in PI [3], [4], [5]P_3_ levels further suggests that it is maintained at the membrane by a dynamic balance between kinase and phosphatase activity, as previously suggested [12].

The ENaC inhibition seen with resveratrol is both rapid in onset and sustained for prolonged periods. To examine the sustained inhibition of ENaC further, we looked for effects of resveratrol on AMPK activity, a known long-term regulator of ENaC activity [39]. We first examined the effect of resveratrol on activation of AMPK, as measured by phosphorylated AMPKα appearance. As shown in **Figure 5**, phosphorylated AMPKα levels increased progressively after 4 h of exposure to resveratrol. To determine whether activated AMPK was involved in the sustained inhibition of ENaC seen with resveratrol, we examined this effect in the presence of AMPK blockade using two distinct techniques. First, we used the inhibitor of AMPK, compound C, and second, we used short-hairpin RNA mediated knockdown of AMPK in mpkCCD_c14_ cells. Given our model that resveratrol inhibits ENaC in the early phase via a mechanism different from AMPK, we would anticipate the inhibitor of AMPK, compound C, would not interfere with the early inhibition. Indeed, as seen in **Figure 6** we observe a stable ENaC current in the control, but in cells pre-treated Compound C followed by resveratrol, the early PI3-K-mediated inhibition remains intact, but is followed by a plateau due to the blockade of the AMPK-dependent effect of resveratrol by Compound C. Based on the same principles, we also would expect to see resveratrol treatment lead to a sustained reduction in current in parental or control shRNA-expressing cells, but that in AMPK shRNA cells, current would plateau, as confirmed in the results seen in **Figure 7C**. With either technique, we saw no effect of AMPK inhibition on the early response to resveratrol, consistent with a primary effect early due to PI3-K inhibition, but a marked amelioration of the sustained inhibition of ENaC seen with resveratrol (**Figures 6** **and** **7C**). These results indicate that the early and late responses to resveratrol may be distinguished between an early PI3-K inhibition affect and a late AMPK-mediated effect.

We propose that the renal and cardioprotective effects of resveratrol described in hypertensive animals may be, to some degree, related to altered Na^+^ handling mediated through inhibition of ENaC by both PI3-K and AMPK pathways. Clearly, other pathways also must be involved, since cardioprotective effects and vascular effects of resveratrol have been described in animal models where hypertension is not affected by this agent [20].
